# Ethyl 2-oxo-3-(3-phthalimidoprop­yl)-2,3-dihydro-1*H*-1,3-benzimidazole-1-carboxyl­ate

**DOI:** 10.1107/S1600536813008325

**Published:** 2013-04-05

**Authors:** Dounia Belaziz, Youssef Kandri Rodi, Adiba Kandri Rodi, El Mokhtar Essassi, Mohamed Saadi, Lahcen El Ammari

**Affiliations:** a Laboratoire de Chimie Organique Appliquée, Université Sidi Mohamed Ben Abdallah, Faculté des Sciences et Techniques, Route d’Immouzzer, BP 2202 Fès, Morocco; b Laboratoire de Chimie Organique Hétérocyclique URAC21, Pôle de Compétences Pharmacochimie, Université Mohammed V-Agdal, Avenue Ibn Battouta, BP 1014 Rabat, Morocco; c Institute of Nanomaterials and Nanotechnology, MASCIR, Rabat, Morocco; d Laboratoire de Chimie du Solide Appliquée, Université Mohammed V-Agdal, Faculté des Sciences, Avenue Ibn Battouta, BP 1014 Rabat, Morocco

## Abstract

In the title compound, C_21_H_19_N_3_O_5_, the phthalimide and benzamidazole ring systems are linked by a propyl chain. The benzamidazole unit also carries an eth­oxy­carbonyl substit­uent. The phthalimido and benzimidazole ring systems are essentially planar, the maximum deviations from their mean planes being 0.008 (2) and 0.020 (2) Å, respectively. The two ring systems are almost orthogonal to one another, making a dihedral angle of 82.37 (8)°. In the crystal, C—H⋯O hydrogen bonds and C—H⋯π contacts stack the mol­ecules along the *b* axis.

## Related literature

For the pharmacological and biochemical properties of benzamidazoles, see: Gravatt *et al.* (1994 ▶); Horton *et al.* (2003 ▶); Kim *et al.* (1996 ▶); Roth *et al.* (1997 ▶); Zarrinmayeh *et al.* (1998 ▶); Spasov *et al.* (1999 ▶). For their use as inter­mediates in many organic reactions, see: Bai *et al.* (2001 ▶); Hasegawa *et al.* (1999 ▶). For their use as ligands to transition metals, see: Bouwman *et al.* (1990 ▶). For a related structure, see: Belaziz *et al.* (2013 ▶).
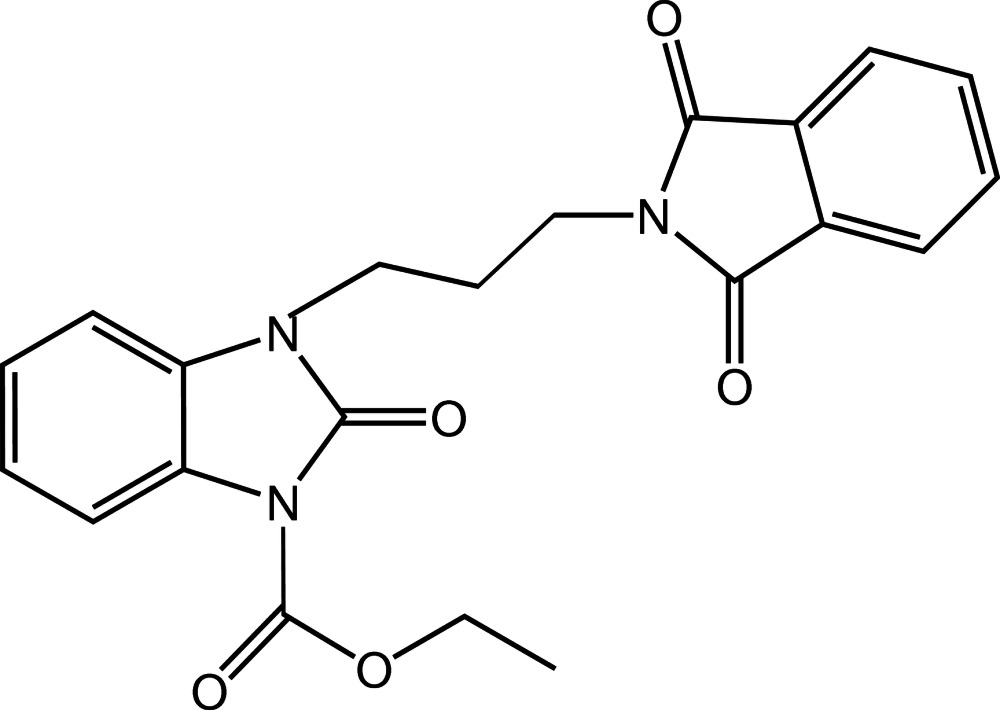


## Experimental

### 

#### Crystal data

C_21_H_19_N_3_O_5_
*M* *_r_* = 393.39
Triclinic, 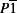

*a* = 5.2850 (7) Å
*b* = 10.6663 (12) Å
*c* = 16.505 (2) Å
α = 86.454 (7)°
β = 83.424 (8)°
γ = 89.376 (7)°
*V* = 922.5 (2) Å^3^
*Z* = 2
Mo *K*α radiation
μ = 0.10 mm^−1^
*T* = 296 K
0.41 × 0.32 × 0.21 mm

#### Data collection

Bruker X8 APEXII area-detector diffractometer
19211 measured reflections
3384 independent reflections
2405 reflections with *I* > 2σ(*I*)
*R*_int_ = 0.037

#### Refinement

*R*[*F* ^2^ > 2σ(*F* ^2^)] = 0.051
*wR*(*F* ^2^) = 0.145
*S* = 1.04
3384 reflections
263 parameters
H-atom parameters constrained
Δρ_max_ = 0.61 e Å^−3^
Δρ_min_ = −0.38 e Å^−3^

### 

Data collection: *APEX2* (Bruker, 2009 ▶); cell refinement: *SAINT* (Bruker, 2009 ▶); data reduction: *SAINT*; program(s) used to solve structure: *SHELXS97* (Sheldrick, 2008 ▶); program(s) used to refine structure: *SHELXL97* (Sheldrick, 2008 ▶); molecular graphics: *ORTEP-3 for Windows* (Farrugia, 2012 ▶); software used to prepare material for publication: *PLATON* (Spek, 2009 ▶) and *publCIF* (Westrip, 2010 ▶).

## Supplementary Material

Crystal structure: contains datablock(s) I, global. DOI: 10.1107/S1600536813008325/sj5310sup1.cif

Structure factors: contains datablock(s) I. DOI: 10.1107/S1600536813008325/sj5310Isup2.hkl

Supplementary material file. DOI: 10.1107/S1600536813008325/sj5310Isup3.cml

Additional supplementary materials:  crystallographic information; 3D view; checkCIF report

## Figures and Tables

**Table 1:** Hydrogen-bond geometry (Å, °) *Cg*1 is the centroid of the C12–C17 ring

*D*—H⋯*A*	*D*—H	H⋯*A*	*D*⋯*A*	*D*—H⋯*A*
C9—H9*A*⋯O2^i^	0.97	2.72	3.523 (3)	141
C13—H13⋯O2^i^	0.93	2.67	3.375 (3)	133
C21—H21*B*⋯O3^ii^	0.96	2.67	3.261 (4)	121
C11—H11*A*⋯O1^iii^	0.97	2.68	3.201 (3)	114
C5—H5⋯*Cg*1^iv^	0.93	2.91	3.750 (3)	151

Symmetry codes: (i) 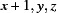; (ii) 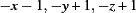; (iii) 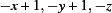; (iv) 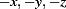.

**Fig. 1.**
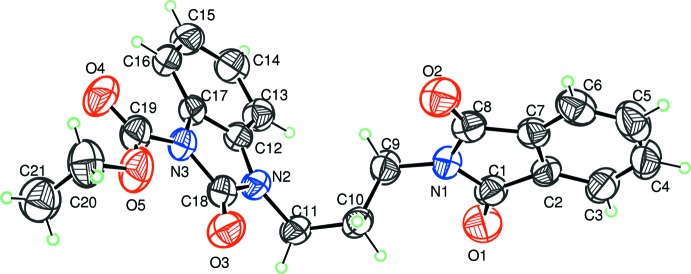
Molecular structure of the title compound with the atom-labelling scheme. Displacement ellipsoids are drawn at the 50% probability level. H atoms are represented as small circles.

**Fig. 2.**
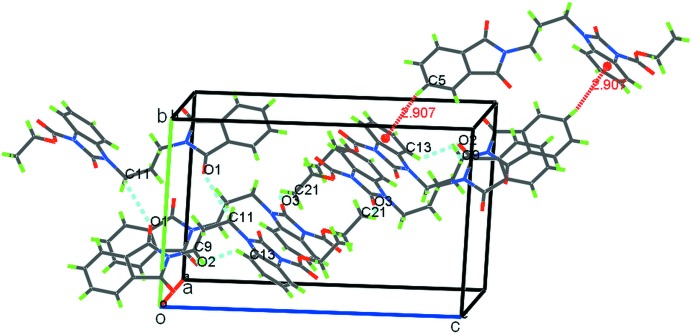
Crystal packing for the title compound viewed along the a axis. C–H,,,O hydrogen bonds are shown as blue dashed lines with the C–H···π contacts shown as dashed red lines, The red spheres represent the centroids of the C12···C17 rings.

## supplementary crystallographic information

### Comment

The benzimidazole nucleus is of significant importance in medicinal chemistry. Several publications report benzimidazole-containing compounds showing biological activities (Zarrinmayeh *et al.*, 1998). Substituted benzimidazole derivatives have found commercial applications in veterinary medicine as anthelmintic agents (Spasov *et al.*, 1999). Functionalized benzimidazoles represent an important class of N-containing heterocyclic compounds and have received considerable attention in recent times because of their applications as antiulcer, antihypertensive, antiviral, antifungal, anticancer and antihistamine agents, among others (Gravatt *et al.*, 1994; Horton *et al.*, 2003; Kim *et al.*, 1996; Roth *et al.*, 1997). They are important intermediates in many organic reactions (Bai *et al.*, 2001; Hasegawa *et al.*, 1999) and act as ligands to transition metals for modelling biological systems (Bouwman *et al.*, 1990). Owing to the potential biological and other technical interest of the benzimidazole family of compounds, a number of synthetic strategies have been developed for the preparation of substituted benzimidazoles.

As a continuation of our research into the development of substituted benzimidazol-2-one derivatives (Belaziz *et al.*, 2013) we report here the synthesis of a new benzimidazol-2-one derivative by the reaction of *N*-(3-bromopropyl)phthalimide with 1-ethoxycarbonyl-benzo[*d*]imidazol-2(3*H*)-one using the same conditions to produce the title compound (Scheme 1).

The crystal structure of the title compound is built up from two fused five and six-membered rings (N1C1 to C8) and (N2N3C12 to C18) linked to a (C9 to C11) chain and one of them is linked to an ethoxycarbonyl group (Fig.1). The fused-ring systems are essentially planar, with the maximum deviation of 0.008 (2) Å for C2 atom and 0.020 (2) Å for C14. The dihedral angle between the phthalimido and the benzo[*d*]imidazol cycles is 82.37 (8)°.

In the crystal structure C21–H21B···O3 and C11–H11···O3 hydrogen bonds form inversion dimers while O2 acts as a bifurcated acceptor forming C9–H9A···O2 and C13–H13···O2 hydrogen bonds, Table 1. These combine with C5–H5···Cg1 contacts to stack molecules along the *b* axis, Fig. 2.

### Experimental

To 1-ethoxycarbonyl-benzo[*d*]imidazol-2(3*H*)-one (0.2 g, 0.97 mmol), potassium carbonate (0.28 g, 1.94 mmol) and tetra-n-butylammonium bromide (0.03 g, 0.1 mmol) in DMF (20 ml) was added *N*-(3-bromopropyl)phthalimide (0.28 g, 1.07 mmol). Stirring was continued at room temperature for 6 h. The salt was removed by filtration and the filtrate concentrated under reduced pressure. The residue was separated by chromatography on a column of silica gel with ethyl acetate/hexane (1/2) as eluent. Crystals were isolated when the solvent was allowed to evaporate.

### Refinement

All H atoms could be located in a difference Fourier map and were treated as riding with C—H = 0.93 Å (aromatic), C—H = 0.97 Å (methylene) and C—H = 0.96 Å methyl. *U*_iso_(H) = 1.2 *U*_eq_(aromatic, methylene) and *U*_iso_(H) = 1.5 *U*_eq_ (methyl).

### Crystal data

**Table:**

C_21_H_19_N_3_O_5_	*Z* = 2
*M**_r_* = 393.39	*F*(000) = 412
Triclinic, *P*1	*D*_x_ = 1.416 Mg m^−^^3^
Hall symbol: -P 1	Mo *K*α radiation, λ = 0.71073 Å
*a* = 5.2850 (7) Å	Cell parameters from 3384 reflections
*b* = 10.6663 (12) Å	θ = 1.2–25.4°
*c* = 16.505 (2) Å	µ = 0.10 mm^−^^1^
α = 86.454 (7)°	*T* = 296 K
β = 83.424 (8)°	Block, colourless
γ = 89.376 (7)°	0.41 × 0.32 × 0.21 mm
*V* = 922.5 (2) Å^3^	

### Data collection

**Table:**

Bruker X8 APEXII area-detector diffractometer	2405 reflections with *I* > 2σ(*I*)
Radiation source: fine-focus sealed tube	*R*_int_ = 0.037
Graphite monochromator	θ_max_ = 25.4°, θ_min_ = 1.2°
φ and ω scans	*h* = −6→6
19211 measured reflections	*k* = −12→12
3384 independent reflections	*l* = −19→19

### Refinement

**Table:**

Refinement on *F*^2^	Secondary atom site location: difference Fourier map
Least-squares matrix: full	Hydrogen site location: inferred from neighbouring sites
*R*[*F*^2^ > 2σ(*F*^2^)] = 0.051	H-atom parameters constrained
*wR*(*F*^2^) = 0.145	*w* = 1/[σ^2^(*F*_o_^2^) + (0.0621*P*)^2^ + 0.3912*P*] where *P* = (*F*_o_^2^ + 2*F*_c_^2^)/3
*S* = 1.04	(Δ/σ)_max_ < 0.001
3384 reflections	Δρ_max_ = 0.61 e Å^−^^3^
263 parameters	Δρ_min_ = −0.38 e Å^−^^3^
0 restraints	Extinction correction: *SHELXL97* (Sheldrick, 2008), Fc^*^=kFc[1+0.001xFc^2^λ^3^/sin(2θ)]^-1/4^
Primary atom site location: structure-invariant direct methods	Extinction coefficient: 0.012 (3)

### Special details

**Table:**

Geometry. All e.s.d.'s (except the e.s.d. in the dihedral angle between two l.s. planes) are estimated using the full covariance matrix. The cell e.s.d.'s are taken into account individually in the estimation of e.s.d.'s in distances, angles and torsion angles; correlations between e.s.d.'s in cell parameters are only used when they are defined by crystal symmetry. An approximate (isotropic) treatment of cell e.s.d.'s is used for estimating e.s.d.'s involving l.s. planes.
Refinement. Refinement of *F*^2^ against all reflections. The weighted *R*-factor *wR* and goodness of fit *S* are based on *F*^2^, conventional *R*-factors *R* are based on *F*, with *F* set to zero for negative *F*^2^. The threshold expression of *F*^2^ > σ(*F*^2^) is used only for calculating *R*-factors(gt) *etc*. and is not relevant to the choice of reflections for refinement. *R*-factors based on *F*^2^ are statistically about twice as large as those based on *F*, and *R*- factors based on all data will be even larger.

### Fractional atomic coordinates and isotropic or equivalent isotropic displacement parameters (Å^2^)

**Table:**

	*x*	*y*	*z*	*U*_iso_*/*U*_eq_	
C5	−0.2365 (7)	0.1185 (3)	−0.2119 (2)	0.0843 (10)	
H5	−0.3437	0.0758	−0.2415	0.101*	
C21	−0.3239 (7)	0.4211 (3)	0.6009 (2)	0.0908 (10)	
H21B	−0.4152	0.4241	0.6545	0.136*	
H21A	−0.3239	0.5030	0.5734	0.136*	
H21C	−0.1516	0.3946	0.6055	0.136*	
N2	0.2008 (4)	0.34071 (17)	0.26388 (11)	0.0470 (5)	
N3	0.0386 (4)	0.26952 (18)	0.38811 (11)	0.0523 (5)	
N1	0.1200 (4)	0.24930 (18)	0.00965 (11)	0.0510 (5)	
C12	0.3548 (4)	0.2414 (2)	0.28783 (13)	0.0443 (5)	
C2	0.0804 (5)	0.2455 (2)	−0.12718 (14)	0.0500 (6)	
O3	−0.1555 (4)	0.44303 (17)	0.32188 (11)	0.0689 (5)	
C17	0.2542 (4)	0.1945 (2)	0.36512 (13)	0.0469 (6)	
C10	0.1307 (5)	0.3982 (2)	0.11910 (14)	0.0507 (6)	
H10A	−0.0524	0.3959	0.1337	0.061*	
H10B	0.1674	0.4644	0.0765	0.061*	
C7	−0.1005 (4)	0.1658 (2)	−0.08645 (15)	0.0506 (6)	
O5	−0.2966 (4)	0.3344 (2)	0.47224 (11)	0.0810 (6)	
O4	−0.0387 (4)	0.1848 (2)	0.51828 (11)	0.0833 (6)	
C8	−0.0771 (5)	0.1668 (2)	0.00225 (15)	0.0522 (6)	
O2	−0.1989 (4)	0.10977 (18)	0.05886 (12)	0.0750 (6)	
C18	0.0058 (5)	0.3619 (2)	0.32393 (14)	0.0520 (6)	
C1	0.2254 (5)	0.3000 (2)	−0.06647 (15)	0.0526 (6)	
C11	0.2575 (5)	0.4292 (2)	0.19332 (13)	0.0504 (6)	
H11A	0.4404	0.4313	0.1785	0.060*	
H11B	0.2036	0.5125	0.2088	0.060*	
C16	0.3660 (5)	0.0934 (2)	0.40379 (15)	0.0582 (7)	
H16	0.2968	0.0601	0.4548	0.070*	
O1	0.4014 (4)	0.37287 (19)	−0.07584 (12)	0.0772 (6)	
C19	−0.0985 (6)	0.2572 (3)	0.46572 (16)	0.0624 (7)	
C9	0.2183 (5)	0.2738 (2)	0.08601 (15)	0.0572 (6)	
H9A	0.4030	0.2727	0.0774	0.069*	
H9B	0.1645	0.2068	0.1265	0.069*	
C14	0.6862 (5)	0.0914 (2)	0.28759 (17)	0.0619 (7)	
H14	0.8350	0.0565	0.2626	0.074*	
C13	0.5705 (5)	0.1904 (2)	0.24783 (15)	0.0534 (6)	
H13	0.6362	0.2215	0.1959	0.064*	
C3	0.1069 (6)	0.2628 (3)	−0.21100 (16)	0.0692 (8)	
H3	0.2295	0.3166	−0.2386	0.083*	
C15	0.5848 (5)	0.0436 (2)	0.36365 (17)	0.0646 (7)	
H15	0.6653	−0.0238	0.3886	0.078*	
C6	−0.2639 (5)	0.1006 (3)	−0.1276 (2)	0.0709 (8)	
H6	−0.3871	0.0471	−0.1000	0.085*	
C4	−0.0562 (7)	0.1970 (3)	−0.25264 (19)	0.0814 (10)	
H4	−0.0426	0.2064	−0.3094	0.098*	
C20	−0.4465 (6)	0.3326 (4)	0.55469 (18)	0.0879 (10)	
H20A	−0.6216	0.3576	0.5500	0.105*	
H20B	−0.4458	0.2490	0.5814	0.105*	

### Atomic displacement parameters (Å^2^)

**Table:**

	*U*^11^	*U*^22^	*U*^33^	*U*^12^	*U*^13^	*U*^23^
C5	0.092 (2)	0.084 (2)	0.088 (2)	0.0255 (19)	−0.044 (2)	−0.0318 (19)
C21	0.089 (2)	0.099 (2)	0.083 (2)	0.0039 (19)	−0.0046 (18)	−0.0065 (19)
N2	0.0562 (11)	0.0444 (10)	0.0386 (10)	−0.0012 (9)	0.0001 (9)	0.0030 (8)
N3	0.0660 (13)	0.0537 (12)	0.0354 (10)	−0.0038 (10)	0.0003 (9)	0.0002 (9)
N1	0.0568 (12)	0.0541 (11)	0.0418 (11)	0.0000 (9)	−0.0056 (9)	−0.0010 (9)
C12	0.0512 (13)	0.0402 (12)	0.0427 (12)	−0.0087 (10)	−0.0106 (10)	−0.0005 (9)
C2	0.0597 (15)	0.0449 (12)	0.0456 (13)	0.0100 (11)	−0.0090 (11)	−0.0001 (10)
O3	0.0777 (13)	0.0662 (12)	0.0581 (11)	0.0161 (10)	0.0075 (9)	0.0029 (9)
C17	0.0591 (14)	0.0431 (12)	0.0399 (12)	−0.0089 (11)	−0.0103 (10)	−0.0029 (10)
C10	0.0571 (14)	0.0498 (13)	0.0427 (13)	0.0048 (11)	−0.0006 (11)	0.0054 (10)
C7	0.0503 (13)	0.0463 (13)	0.0561 (14)	0.0097 (11)	−0.0084 (11)	−0.0066 (11)
O5	0.0870 (15)	0.1053 (16)	0.0465 (11)	0.0083 (13)	0.0089 (10)	−0.0017 (10)
O4	0.1133 (17)	0.0836 (14)	0.0472 (11)	0.0033 (12)	0.0056 (11)	0.0141 (10)
C8	0.0491 (14)	0.0495 (13)	0.0551 (15)	0.0054 (11)	0.0044 (11)	−0.0007 (11)
O2	0.0758 (13)	0.0763 (13)	0.0664 (12)	−0.0086 (10)	0.0165 (10)	0.0047 (10)
C18	0.0630 (15)	0.0501 (13)	0.0418 (13)	−0.0022 (12)	−0.0014 (11)	−0.0025 (10)
C1	0.0587 (15)	0.0473 (13)	0.0498 (14)	0.0003 (12)	−0.0006 (11)	0.0021 (11)
C11	0.0627 (15)	0.0427 (12)	0.0434 (13)	−0.0030 (11)	0.0013 (11)	0.0038 (10)
C16	0.0827 (19)	0.0492 (14)	0.0447 (13)	−0.0104 (13)	−0.0186 (13)	0.0035 (11)
O1	0.0802 (13)	0.0746 (13)	0.0742 (13)	−0.0261 (11)	0.0018 (10)	0.0006 (10)
C19	0.0784 (19)	0.0636 (16)	0.0436 (14)	−0.0090 (14)	−0.0001 (13)	−0.0002 (13)
C9	0.0647 (16)	0.0600 (15)	0.0483 (14)	0.0081 (12)	−0.0134 (12)	−0.0038 (11)
C14	0.0581 (16)	0.0607 (16)	0.0689 (18)	0.0043 (12)	−0.0151 (13)	−0.0067 (13)
C13	0.0532 (14)	0.0545 (14)	0.0521 (14)	−0.0036 (11)	−0.0048 (11)	−0.0010 (11)
C3	0.090 (2)	0.0646 (17)	0.0515 (16)	0.0143 (15)	−0.0071 (15)	0.0041 (13)
C15	0.0774 (19)	0.0518 (15)	0.0684 (18)	0.0036 (13)	−0.0271 (15)	−0.0007 (13)
C6	0.0623 (17)	0.0625 (17)	0.091 (2)	0.0050 (13)	−0.0164 (15)	−0.0170 (15)
C4	0.114 (3)	0.079 (2)	0.0558 (17)	0.031 (2)	−0.0304 (18)	−0.0124 (16)
C20	0.087 (2)	0.113 (3)	0.0630 (19)	−0.005 (2)	0.0007 (17)	−0.0131 (18)

### Geometric parameters (Å, º)

**Table:**

C5—C4	1.365 (5)	C10—H10B	0.9700
C5—C6	1.383 (4)	C7—C6	1.377 (4)
C5—H5	0.9300	C7—C8	1.484 (3)
C21—C20	1.452 (4)	O5—C19	1.324 (3)
C21—H21B	0.9600	O5—C20	1.493 (3)
C21—H21A	0.9600	O4—C19	1.193 (3)
C21—H21C	0.9600	C8—O2	1.208 (3)
N2—C18	1.373 (3)	C1—O1	1.209 (3)
N2—C12	1.394 (3)	C11—H11A	0.9700
N2—C11	1.458 (3)	C11—H11B	0.9700
N3—C19	1.397 (3)	C16—C15	1.383 (4)
N3—C17	1.415 (3)	C16—H16	0.9300
N3—C18	1.426 (3)	C9—H9A	0.9700
N1—C8	1.391 (3)	C9—H9B	0.9700
N1—C1	1.394 (3)	C14—C15	1.377 (4)
N1—C9	1.456 (3)	C14—C13	1.383 (3)
C12—C13	1.374 (3)	C14—H14	0.9300
C12—C17	1.390 (3)	C13—H13	0.9300
C2—C7	1.374 (3)	C3—C4	1.384 (4)
C2—C3	1.376 (3)	C3—H3	0.9300
C2—C1	1.478 (3)	C15—H15	0.9300
O3—C18	1.209 (3)	C6—H6	0.9300
C17—C16	1.381 (3)	C4—H4	0.9300
C10—C9	1.514 (3)	C20—H20A	0.9700
C10—C11	1.517 (3)	C20—H20B	0.9700
C10—H10A	0.9700		
			
C4—C5—C6	121.8 (3)	O1—C1—C2	130.4 (2)
C4—C5—H5	119.1	N1—C1—C2	106.0 (2)
C6—C5—H5	119.1	N2—C11—C10	114.17 (19)
C20—C21—H21B	109.5	N2—C11—H11A	108.7
C20—C21—H21A	109.5	C10—C11—H11A	108.7
H21B—C21—H21A	109.5	N2—C11—H11B	108.7
C20—C21—H21C	109.5	C10—C11—H11B	108.7
H21B—C21—H21C	109.5	H11A—C11—H11B	107.6
H21A—C21—H21C	109.5	C17—C16—C15	117.4 (2)
C18—N2—C12	111.02 (18)	C17—C16—H16	121.3
C18—N2—C11	121.51 (19)	C15—C16—H16	121.3
C12—N2—C11	126.41 (19)	O4—C19—O5	125.7 (3)
C19—N3—C17	122.5 (2)	O4—C19—N3	122.5 (3)
C19—N3—C18	127.8 (2)	O5—C19—N3	111.7 (2)
C17—N3—C18	109.47 (18)	N1—C9—C10	113.48 (19)
C8—N1—C1	111.4 (2)	N1—C9—H9A	108.9
C8—N1—C9	125.0 (2)	C10—C9—H9A	108.9
C1—N1—C9	123.5 (2)	N1—C9—H9B	108.9
C13—C12—C17	121.4 (2)	C10—C9—H9B	108.9
C13—C12—N2	130.5 (2)	H9A—C9—H9B	107.7
C17—C12—N2	108.1 (2)	C15—C14—C13	121.0 (3)
C7—C2—C3	121.3 (3)	C15—C14—H14	119.5
C7—C2—C1	108.5 (2)	C13—C14—H14	119.5
C3—C2—C1	130.2 (3)	C12—C13—C14	117.6 (2)
C16—C17—C12	120.9 (2)	C12—C13—H13	121.2
C16—C17—N3	132.8 (2)	C14—C13—H13	121.2
C12—C17—N3	106.32 (19)	C2—C3—C4	117.4 (3)
C9—C10—C11	112.89 (19)	C2—C3—H3	121.3
C9—C10—H10A	109.0	C4—C3—H3	121.3
C11—C10—H10A	109.0	C14—C15—C16	121.6 (2)
C9—C10—H10B	109.0	C14—C15—H15	119.2
C11—C10—H10B	109.0	C16—C15—H15	119.2
H10A—C10—H10B	107.8	C7—C6—C5	116.9 (3)
C2—C7—C6	121.6 (3)	C7—C6—H6	121.5
C2—C7—C8	107.9 (2)	C5—C6—H6	121.5
C6—C7—C8	130.5 (3)	C5—C4—C3	121.1 (3)
C19—O5—C20	115.4 (2)	C5—C4—H4	119.4
O2—C8—N1	124.7 (2)	C3—C4—H4	119.4
O2—C8—C7	129.2 (2)	C21—C20—O5	106.3 (3)
N1—C8—C7	106.2 (2)	C21—C20—H20A	110.5
O3—C18—N2	126.6 (2)	O5—C20—H20A	110.5
O3—C18—N3	128.4 (2)	C21—C20—H20B	110.5
N2—C18—N3	105.1 (2)	O5—C20—H20B	110.5
O1—C1—N1	123.6 (2)	H20A—C20—H20B	108.7

### Hydrogen-bond geometry (Å, º)

Cg1 is the centroid of the C12–C17 ring

**Table:**

*D*—H···*A*	*D*—H	H···*A*	*D*···*A*	*D*—H···*A*
C9—H9*A*···O2^i^	0.97	2.72	3.523 (3)	141
C13—H13···O2^i^	0.93	2.67	3.375 (3)	133
C21—H21*B*···O3^ii^	0.96	2.67	3.261 (4)	121
C11—H11*A*···O1^iii^	0.97	2.68	3.201 (3)	114
C5—H5···*Cg*1^iv^	0.93	2.91	3.750 (3)	151

Symmetry codes: (i) *x*+1, *y*, *z*; (ii) −*x*−1, −*y*+1, −*z*+1; (iii) −*x*+1, −*y*+1, −*z*; (iv) −*x*, −*y*, −*z*.
